# A lipidomic based metabolic age score for monitoring the effects of lifestyle and diet on metabolic disease risk

**DOI:** 10.21203/rs.3.rs-9239578/v1

**Published:** 2026-06-09

**Authors:** Tingting Wang, Habtamu B Beyene, Changyu Yi, Thy Duong, Natalie A Mellett, Thomas G Meikle, Jingqin Wu, Aleksandar Dakic, Michelle Cinel, Gerald F Watts, Joseph Hung, Jennie Hui, John Beilby, John Blangero, Agus Salim, Eric K Moses, Matthias Arnold, Gabi Kastenmüller, Xianlin Han, Colette Blach, Chenglong Yu, John J. McNeil, Paul Lacaze, Kwangsik Nho, Andrew J Saykin, Jonathan E Shaw, Dianna J Magliano, Alzheimer’s Disease Neuroimaging Initiative, Rima Kaddurah-Daouk, Corey Giles, Kevin Huynh, Peter J Meikle

**Affiliations:** 1 Baker Heart and Diabetes Institute, Melbourne, Australia.; 2 Baker Department of Cardiometabolic Health, Melbourne University, Melbourne, Australia.; 3 Baker Department of Cardiovascular Research Translation and Implementation, La Trobe University, Melbourne, Australia.; 4 School of Medicine, University of Western Australia, Perth, Australia.; 5 Lipid Disorders Clinic, Department of Cardiology, Royal Perth Hospital, Perth, Australia.; 6 PathWest Laboratory Medicine of WA, Nedlands, WA 6009; 7 School of Population and Global Health, University of Western Australia, Crawley, Western Australia.; 8 School of Biomedical Sciences, University of Western Australia, Nedlands, Western Australia.; 9 South Texas Diabetes and Obesity Institute, The University of Texas Rio Grande Valley, Brownsville, Texas, USA.; 10 Melbourne School of Population and Global Health and School of Mathematics and Statistics, The University of Melbourne; 11 Menzies Institute for Medical Research, University of Tasmania, Hobart, Tasmania, Australia.; 12 Department of Psychiatry and Behavioral Sciences, Duke University, Durham, NC, USA; 13 Institute of Computational Biology, Helmholtz Zentrum München, German Research Center for Environmental Health, Neuherberg, Germany; 14 Barshop Institute for Longevity and Aging Studies, University of Texas Health Science Center at San Antonio, San Antonio, TX, USA; 15 Duke Molecular Physiology Institute, Duke University, Durham, NC, USA.; 16 School of Public Health and Preventive Medicine, Monash University, Melbourne, Victoria, Australia; 17 Department of Radiology and Imaging Sciences, Indiana University School of Medicine, Indianapolis IN, USA; 18 Indiana Alzheimer s Disease Research Center, Indiana University School of Medicine, Indianapolis, IN, USA.; 19 Duke Institute of Brain Sciences, Duke University, Durham, NC, USA; 20 Department of Medicine, Duke University, Durham, NC, USA

## Abstract

Biological age scores capture ageing heterogeneity beyond chronological age but are often dominated by lifestyle and environmental exposures, limiting clinical interpretability. We developed an environmentally adjusted metabolic age score (EAmAge) to isolate intrinsic ageing biology relevant to neurodegeneration and chronic disease. Major environmental influences were statistically removed from plasma lipidomic profiles before constructing an age-prediction model using ridge regression. EAmAge was derived in the AusDiab cohort (n = 10,339) and validated across three independent cohorts (BHS, ADNI and ASPREE; total n = 9,835). Compared with an unadjusted lipidomic age model (mAge_orig), EAmAge showed stronger and more consistent associations with incident Alzheimer s disease-related dementia, cardiovascular events and all-cause mortality. EAmAge was also associated with Alzheimer s disease-related biomarkers, including amyloid burden, reduced glucose metabolism and hippocampal atrophy. These findings establish EAmAge as a robust and partially modifiable biomarker that improves risk stratification by disentangling intrinsic metabolic ageing from environmental confounding.

## Introduction

Chronological age is a major risk factor for many chronic diseases, driving both morbidity and mortality. With ageing, many physiological processes are slowing down, leading to increased risk of age-related diseases. However, individuals of the same chronological age can vary markedly in their underlying risk of age-related diseases^1,2^. Their biological age, shaped by differences in genetic predisposition, metabolic adaptability, and life-course exposures^3–5^ can dramatically influence resilience to the ageing process. Many techniques have emerged to estimate the underlying biological age, utilising physiological and molecular changes to estimate health span^6^. Accelerated biological aging has been linked to a higher risk of heart disease, diabetes, cancer and neurodegenerative diseases, ultimately leading to increased morbidity and mortality^7–13^. Biological age provides the opportunity to identify individuals at increased risk of age-related disease and the potential to monitor response to interventions aimed at slowing the ageing process.

Recent advances in large-scale omics profiling have enabled the development of molecular ageing biomarkers that quantify deviations from chronological ageing. Of these, metabolic ageing^13–17^, derived from metabolomic data, have gained attention as indicators of age-related physiological changes. Driven by strong evidence of the intimate link between the lipidome and age^18–20^, we recently reported a comprehensive lipidome based metabolic age^13^. The findings suggest that metabolic age can efficiently identify individuals at a higher risk of clinical outcomes, highlighting those who may benefit from early lifestyle or clinical intervention. However, the calculation of metabolic age - as well as other biological age scores - has mostly been done in cross-sectional population datasets. This approach introduces potential confounding because participants born in different calendar years/decades are exposed to varying environmental factors (e.g., diet, lifestyle, disease burden), which can differentially influence lipid metabolism and the lipidome^20^. As a result, conventional metabolic age scores may partially capture environmentally driven variation rather than intrinsic ageing biology. The calculated metabolic age can then be inherently and mathematically confounded by inter-generation dependent exposures^2,20–22^: age-related shifts in diet, smoking, alcohol consumption, and physical activity correlate strongly with chronological age and can be inadvertently embedded within lipidomic predictors. Consequently, associations between metabolic age and clinical outcomes may reflect lifestyle exposure rather than underlying metabolic ageing processes.

To address this, we developed an environmentally adjusted metabolic age score (EAmAge) by explicitly separating lifestyle- and diet-related variation from intrinsic metabolic ageing signals in the plasma lipidome. Using the AusDiab cohort (n = 10,339), we first quantified the component of each lipid species that could be explained by measured environmental factors, including diet and lifestyle, and removed this component to obtain lipid profiles less influenced by environmental exposures. These adjusted lipid profiles were then used to train a penalized ridge regression model to estimate metabolic age. The resulting score was externally validated across three independent population and clinical cohorts (total n = 9,835). Compared with a conventional, unadjusted metabolic age model (mAge_orig), the environmentally adjusted score showed stronger and more consistent associations with incident Alzheimer s disease and related dementias, cardiovascular outcomes, and all-cause mortality, supporting its ability to better capture intrinsic metabolic ageing relevant to long-term disease risk.

## Results

### Development and Validation of Metabolic Age Models

**Figure 1.**
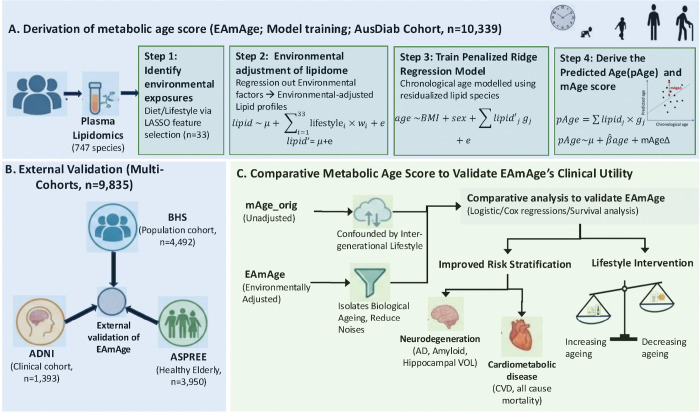
Study design for derivation and validation of the environmentally adjusted metabolic age score (EAmAge). **A.** Derivation of the metabolic age score in the AusDiab cohort. Environmental influences (lifestyle and dietary factors) were regressed out from plasma lipidomic profiles. Ridge regression was then applied to predict chronological age using BMI, sex, and the environmentally adjusted lipidome, and model coefficients were extracted. Metabolic age (mAge) and age acceleration (mAgeΔ) were calculated in validation cohorts by summing lipid abundances weighted by the derived coefficients. **B.** External validation of EAmAge in three independent cohorts: ADNI, BHS, and ASPREE. **C.** Within each cohort, the performance of EAmAge was compared with the unadjusted model (mAge_orig) by evaluating associations with clinical outcomes and environmental factors.

We developed two lipidome-based metabolic age models in the AusDiab cohort: an original version without adjustment for environmental factors (mAge_orig) and an environment-adjusted version (EAmAge) (Figure 1). Both models were trained in AusDiab and externally validated in three independent cohorts: the Busselton Health Study (BHS), the Alzheimer s Disease Neuroimaging Initiative (ADNI), and the Aspirin in Reducing Events in the Elderly (ASPREE) study. Clinical outcomes included prevalent cardiovascular disease (CVD) and incident major cardiovascular events (CVE) in AusDiab, BHS and ASPREE, all-cause mortality in AusDiab and ASPREE, and neurodegenerative outcomes (Alzheimer s disease or dementia) in ADNI and ASPREE (Table 1; Supplementary Figure 1).

Models were fitted using penalised ridge regression with ten-fold cross-validation in AusDiab (Figure 1). The direct model output, predicted age (pAge), represents the chronological age predicted from lipidomic features. To quantify metabolic age acceleration independent of chronological age, we derived metabolic age delta (mAgeΔ) as the residual from regressing pAge on chronological age. We then calculated metabolic age (mAge) as chronological age plus mAgeΔ, enabling interpretation on the age scale while preserving an age-independent acceleration component.

The defining difference between mAge_orig and EAmAge was the treatment of environmental exposures. For mAge_orig, the full lipidome was used directly to predict age. For EAmAge, we removed environmentally attributable lipid variation before age-model training: each lipid species was regressed on measured environmental factors (including diet and lifestyle variables), and the resulting residual lipid values were used as the input features for ridge regression. This workflow ensured that environmental effects were accounted for at the lipid level upstream of model construction, rather than being handled only in downstream association models.

### Predictive performance of metabolic age models across cohorts.

Model performance was determined using the coefficient of determination (*R*^*2*^; the variance in age explained by the lipidome), calculated as the square of the Pearson correlation between pAge and chronological age. In the AusDiab cohort under ten-fold cross validation, the lipidome (without environmental adjustment) could capture 69% of the variance in age (Table 2). After regressing environmental factor information from the lipidome, predictive performance remained similar (*R*^*2*^=0.68) (Supplementary Figure 2).

In the external BHS validation cohort, comparable predictive performance was observed: *R*^*2*^ = 0.71 for the original model and *R*^*2*^ = 0.73 for the model with environmental factors adjustment (Table 2). However, in the two clinical validation cohorts with older age distributions (ASPREE and ADNI), we trained the models using an age-restricted subset of the AusDiab cohort (participants aged ≥60 years). This resulted in a notable decline in predictive performance. Within the subset group of AusDiab, cross-validation yielded *R*^*2*^ values of 0.37 for the original model and 0.21 for the environmentally adjusted model. When applied to the external validation cohorts, pAge exhibited weaker associations with chronological age: in ADNI, *R*^*2*^ was 0.26 for the original model and 0.16 for the adjusted model; in ASPREE, *R*^*2*^ was 0.13 (original) and 0.11 (adjusted). This reduction in model performance is expected, reflecting the narrower age range and greater heterogeneity in health status among older participants.

We additionally examined the correlations of mAge with chronological age. In AusDiab, the *R*^*2*^ value for mAge was 0.82 (original model) and 0.83 (adjusted model). In BHS, these correlations increased to 0.85 (original model) and 0.89 (adjusted model). Full results are reported in Table 2.

Lipid species from acylcarnitine (AC), sphingomyelin (SM), alkenylphosphatidylcholine (PC(P)), lysophosphatidylcholine (LPC), lysoalkylphosphatidylcholine (LPC(O)) were major predictors in both mAge models (Supplementary Table 1–2).

### Metabolic age demonstrated significant confounding by environmental factors.

To evaluate the impact of environmental adjustment on lifestyle and diet associations, we compared two age-independent residual-based metrics termed mAge_origΔ (derived from original model) and EAmAgeΔ (derived from EAmAge). Specifically, mAge_origΔ was defined as the residual from regressing pAge (original model) on chronological age, whereas EAmAgeΔ was defined as the residual from regressing pAge (environmental factor adjustment model) on chronological age.

**Figure 2.**
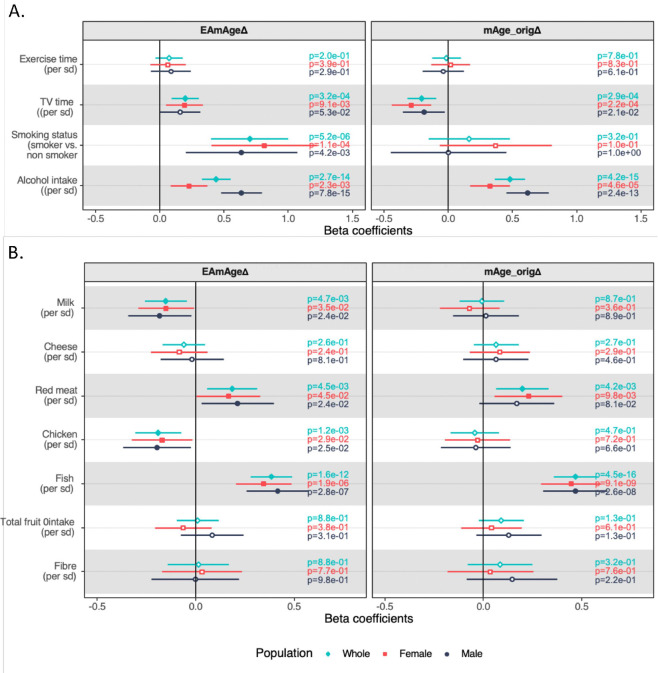
Associations of mAge_origΔ and EAmAgeΔ with environmental factors in the AusDiab cohort. Separate linear regression model were carried out to examine different environmental factors with two sets of mAgeΔ scores with (EAmAgeΔ) or without environmental adjustment (mAge_origΔ). Smoking status (current smoker vs. non-smoker) was treated as a categorical variable. All other continuous environmental factors were inverse rank normalized and are interpreted per standard deviation change.

Smoking and alcohol intake had a small negative association with mAge_origΔ (smoking: *beta*=−0.195, *p*=2.86×10^−01^; alcohol intake: *beta*=−0.158, *p*=2.25×10^−02^). However, in the environmentally adjusted model, both factors were significantly positively associated with higher EAmAgeΔ, (smoking *beta*=0.698, *p*=5.22×10^−06^; alcohol intake *beta*=0.436, *p*=2.69×10^−14^; Figure 2A; Supplementary Table 3). Both mAge_origΔ and EAmAgeΔ exhibited positive associations with TV viewing time, and we note that exercise time did not show a significant impact on either of the metabolic age scores.

Associations with dietary intake also showed some discordant effects between adjusted and non-adjusted models. Red meat intake reversed association direction, becoming positively associated with EAmAgeΔ (*beta*=0.184, *p*=4.53×10^−03^), whereas it was negatively associated in the mAge_origΔ (*beta*=−0.167, *p*=3.91×10^−02^) (Figure 2B; Supplementary Table 3). Intake of milk became significantly associated with lower EAmAgeΔ (beta=−0.154, p=4.68×10^−03^). Higher fish consumption was associated with increased metabolic age in both models, while higher intake of chicken correlated with lower metabolic age across both models.

Moreover, both mAge_origΔ and EAmAgeΔ were consistently and positively associated with omega-3 intake, indicating that higher consumption was linked to increased metabolic age (Supplementary Figure 3; Supplementary Table 3). To assess whether these associations were driven primarily by omega-3 related lipid species within the full lipidome, we conducted sensitivity analyses using models built with feature sets restricted to clinical lipids or acylcarnitine species, neither of which include omega-3 species. Notably, the positive associations with omega-3 intake remained robust, suggesting that the relationship is not solely driven by omega-3 lipid content (Supplementary Figure 4; Supplementary Table 4). Interestingly, mAge_origΔ also demonstrated significant negative associations with monounsaturated fatty acids (MUFA) (beta=−0.685, p=2.47×10^−05^) and odd-chain fatty acids (OCFA) (beta=−0.367, p=7.07×10^−04^) (Supplementary Figure 3; Supplementary Table 3). However, these associations were not observed in the environmentally adjusted model (EAmAgeΔ), indicating that environmental confounders may modulate the lipidomic links with dietary fatty acids.

All observed associations persisted consistently across both male and female populations.

Given the stronger and more consistent exposure associations observed for EAmAgeΔ, we conducted age-stratified analyses comparing participants aged <60 and ≥ 60 years (Supplementary Figure 5–6; Supplementary Table 5). Associations were generally consistent across age groups, with particularly strong positive associations for smoking and alcohol intake in the younger group. Exercise time showed opposite directions by age group, associating with lower EAmAgeΔ in older participants (beta=−0.277, p=9.5×10^−03^) but higher EAmAgeΔ in younger participants (beta=0.191, p=3.79×10^−03^).

### Accounting for environmental factors uncovers links between elevated metabolic age and neurodegenerative disease markers.

**Figure 3.**
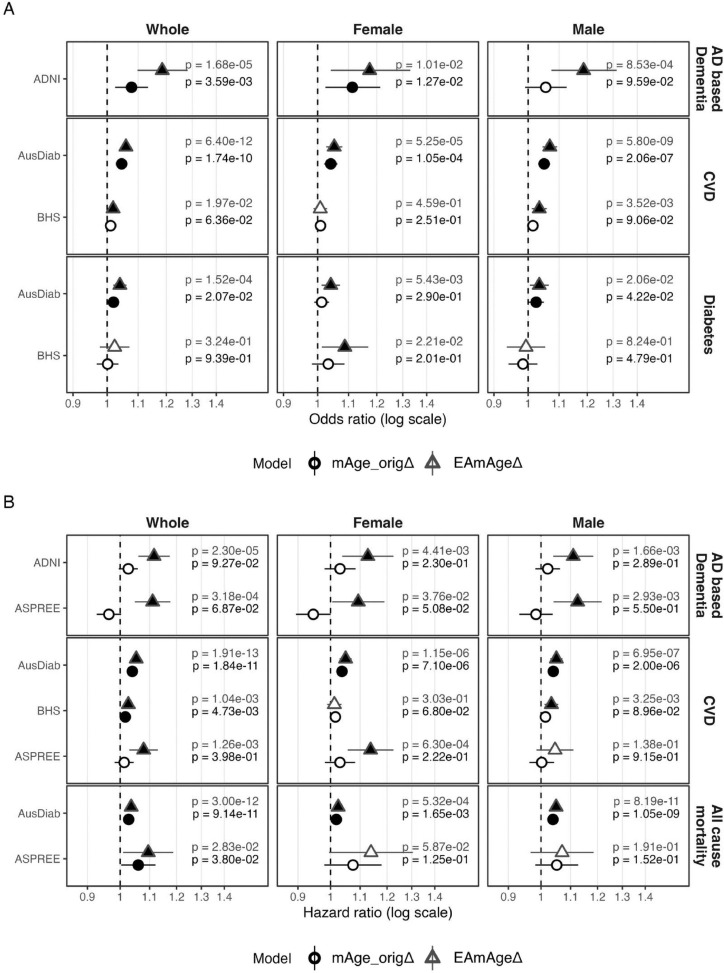
Associations of clinical outcomes with mAge_origΔ and EAmAgeΔ in the sex-stratified population of the AusDiab, BHS, ADNI and ASPREE cohorts. The associations of mAgeΔ from mAge_orig and EAmAge with prevalent outcomes were examined using logistic regression models (A). The associations of mAgeΔ from mAge_orig and EAmAge with incident outcomes were examined using Cox regression models (B).

The two metabolic age models (mAge_orig and EAmAge) were trained using an age-restricted subset of the AusDiab cohort (participants aged ≥ 60 years). The adjustment for environmental factors in the metabolic age models enhanced the associations between metabolic age and neurodegenerative disorder-related diseases or biomarkers. In the ADNI cohort, EAmAgeΔ (*OR*=1.19, *p*=1.68×10^−05^) exhibited significantly stronger associations with prevalent AD cases compared to mAge_origΔ (*OR*=1.08, *p*=3.59×10^−03^) (Figure 3A; Supplementary Table 6). Similarly, relative to mAge_origΔ (*HR*=1.03, *p*=9.27×10^−02^), EAmAgeΔ (*HR*=1.12, *p*=2.30×10^−05^) showed stronger associations with incident AD (Figure 3B; Supplementary Table 7). All results for the AD cases in the ADNI cohort remained consistent across both females and males.

**Figure 4.**
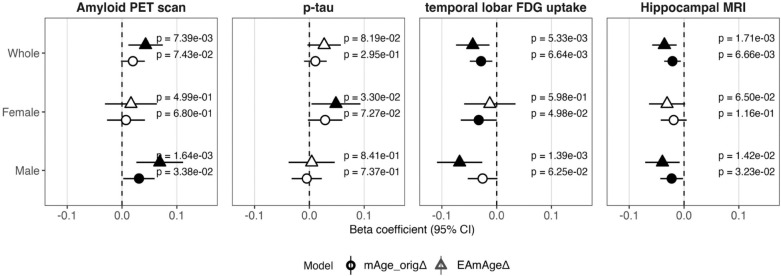
Associations of AD biomarkers with mAge_origΔ and EAmAgeΔ in the ADNI cohort. Separate sets of linear regression models were carried out to examine the associations of age, mAge, and mAgeΔ with AD related biomarkers including amyloid deposition measured from amyloid PET scans (AmyPET, n=742; A), CSF phosphorylated tau (p-tau, n=1,009; B) levels, temporal lobar FDG uptake (FDG_Temp, n=1,059; C), and hippocampal volumn MRI (HippVol_IUSM_FS6, n=1,385; D).

Moreover, we explored the associations of mAgeΔ with AD-based biomarkers, including amyloid PET scan (AmyPet), phosphorylated tau (p-tau), temporal lobe glucose metabolism assessed by FDG-PET (FDG_Temp), and hippocampal volume measured by MRI (HippVol). We observed both versions of mAgeΔ showed moderate improvements in the associations with these biomarkers, independent of age. Notably, for three biomarkers (AmyPet, FDG_Temp, and HippVol), EAmAgeΔ consistently showed slightly greater associations (AmyPet: *beta*=0.043, *p*=7.39×10^−03^; FDG_Temp: *beta*=−0.044, *p*=5.33×10^−03^; HippVol: *beta*=−0.036, *p*=1.71×10^−03^) compared to mAge_origΔ (AmyPet: *beta*=0.020, *p*=7.43×10^−02^; FDG_Temp: *beta*=−0.029, *p*=6.64×10^−03^; HippVol: *beta*=−0.021, *p*=6.66×10^−03^) (Figure 4; Supplementary Table 8). However, for p-Tau, neither version of mAgeΔ presented any significant associations. Biomarker associations were consistent in males; in females, several associations attenuated, likely reflecting reduced sample size.

Additionally, we validated the associations with incident dementia in the ASPREE cohort. We observed that EAmAgeΔ demonstrated a significant association with incident dementia, whereas mAge_origΔ showed no significant associations. (Figure 3B; Supplementary Table 7).

### Environment adjusted metabolic age has stronger associations with CVD and all-cause mortality risk.

Because CVD outcomes span a wide age range, we trained models in the full AusDiab cohort. Both mAge_origΔ and EAmAgeΔ, were associated with CVD outcomes, independent of age (Figure 3; Supplementary Table 6–7). We conducted logistic regression to examine the associations of mAge and mAgeΔ with prevalent CVD, adjusted for sex and BMI. Relative to mAge_origΔ (*OR*=1.05, *p*=1.74×10^−10^), EAmAgeΔ (*OR*=1.06, *p*=6.4×10^−12^) showed stronger associations with prevalent CVD. Similarly, for the incident CVD within a 12-year follow-up period, Cox regression showed enhanced associations of EAmAgeΔ (*HR*=1.06, *p*=1.91×10^−13^) compared to mAge_origΔ (*HR*=1.04, *p*=1.84×10^−11^).

These associations were validated in the BHS cohort, where both metrics remained significantly associated with prevalent and incident CVD over 20 years of follow-up, with comparable effect sizes (Figure 3; Supplementary Table 6–7).

For mortality in AusDiab (17-year follow-up), both metrics were associated with all-cause mortality independent of age, with stronger evidence for EAmAgeΔ (EAmAgeΔ*: HR*=1.04, *p*=3.00×10^−12^; mAge_origΔ: *HR*=1.03, *p*=9.14×10^−11^), consistent across sexes (Figure 3B, Supplementary Table 7).

In ASPREE, EAmAgeΔ was associated with future CVD (HR=1.08, p=1.26×10^−03^), whereas mAge_origΔ was not (HR=1.01, p=3.98×10^−01^). Both metrics were associated with mortality in ASPREE with similar effect magnitudes (Figure 3B, Supplementary Table 7).

### Survival analyses using EAmAgeΔ deciles.

**Figure 5.**
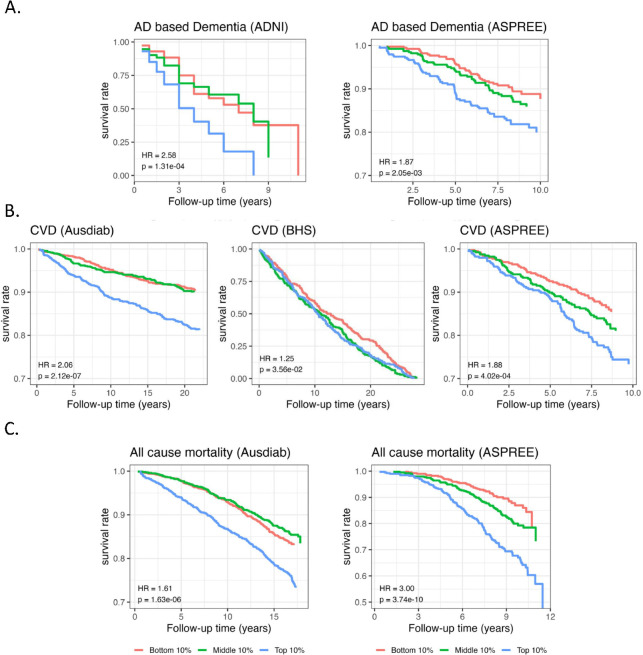
Survival analyses stratified by EAmAgeΔ. Kaplan–Meier survival curves for (A) dementia-free survival, (B) incident cardiovascular disease, and (C) all-cause mortality across independent cohorts - ADNI, ASPREE, AusDiab, BHS, and ASPREE. Participants were stratified into deciles of environmentally adjusted metabolic age (**EAmAgeΔ**), with curves shown for the lowest decile (bottom 10%), middle decile, and highest decile (top 10%). Hazard ratios (HRs) and p values were derived from Cox proportional hazards models.

Participants were stratified into deciles of EAmAgeΔ, and Kaplan–Meier survival curves compared the lowest decile (bottom 10%), middle decile, and highest decile (top 10%). Higher EAmAgeΔ was associated with reduced survival across outcomes and cohorts. For AD related dementia, individuals in the highest EAmAgeΔ decile exhibited significantly lower dementia-free survival compared with the lowest decile in both ADNI (HR=2.58, *p*=1.31×10^−04^) and ASPREE (HR=1.87, *p*=2.05×10^−03^) (Figure 5A). Elevated EAmAgeΔ was similarly associated with higher risk of incident cardiovascular disease in AusDiab (HR=2.06, *p*=2.12×10^−07^), BHS (HR=1.25, *p*=3.56×10^−02^), and ASPREE (HR=1.88, *p*=4.02×10^−04^) (Figure 5B). Consistent patterns were observed for all-cause mortality, with higher EAmAgeΔ associated with lower survival in AusDiab (HR=1.61, *p*=1.63×10^−06^) and ASPREE (HR=3.00, *p*=3.74×10^−10^) (Figure 5C). Across all outcomes, participants in the middle EAmAgeΔ decile exhibited intermediate survival probabilities, consistent with a graded association between metabolic ageing and long-term clinical risk.

## Discussion

Driven by the goal of understanding the molecular changes underlying the aging process, various measurements of biological age^6^, based on transcriptomics^23–25^, proteomics^12,26–28^, and more recently, metabolomics^14–17^, have been introduced. Many such models are derived from cross-sectional cohorts, where lifestyle and dietary behaviours measured at the time of sampling are strongly correlated with chronological age. As a result, age-associated environmental exposures may be implicitly embedded within omics-based ageing signatures, complicating interpretation of downstream disease associations. In this study, we demonstrate that explicitly accounting for contemporaneous diet- and lifestyle-related lipidomic variation improves the ability of a metabolic ageing model to capture risk of age-related diseases. Using a large population-based cohort (AusDiab, n=10,339) for model development and validating across three independent cohorts (BHS, ADNI, and ASPREE; total n = 9,835), we show that an environmentally adjusted metabolic age score exhibits stronger and more consistent associations with Alzheimer s disease, cardiovascular disease, and mortality than a conventional unadjusted model. This is supported by our previous lipidomic studies that highlight lipid associations with Alzheimer s disease^29–31^ and cardiovascular disease^13,32^ independent of many common confounders and chronological age.

This adjustment revealed biologically plausible associations between metabolic ageing and lifestyle factors. Smoking and alcohol intake can accelerate an individual s metabolic aging, whereas higher consumption of milk, and chicken can reduce metabolic age. Another interesting finding was that excessive red meat intake could increase metabolic age (after controlling for environmental factors), aligning with evidence linking high red meat consumption to adverse metabolic and cardiovascular outcomes. Compared with mAge_origΔ, EAmAgeΔ showed stronger and more consistent associations with these behaviours, suggesting that removing environmental signal during score derivation enhanced sensitivity to lifestyle-related metabolic variation. Exercise demonstrated age-dependent associations, with higher activity linked to lower metabolic age in participants aged ≥60 years but higher metabolic age in those aged <60 years, potentially reflecting improved cardiometabolic resilience in older adults versus greater metabolic demands or residual confounding in younger individuals. Given the observational design, these findings should be interpreted cautiously and warrant longitudinal validation but collectively suggest that EAmAge may provide a more behaviourally responsive metric of metabolic ageing.

Unexpectedly, we observed a positive association between omega-3 polyunsaturated fatty acids (n-3 PUFAs) dietary intake and both versions of the metabolic age score (mAge_origΔ and EAmAgeΔ). This association persisted across several sensitivity analyses, including models limited to either clinical lipids or acylcarnitine species - both of which excluded omega-3-related lipids yet still demonstrated consistent positive associations. These findings stand in contrast to the well-established cardioprotective and anti-inflammatory roles of eicosapentaenoic acid (EPA) and docosahexaenoic acid (DHA), which are known to reduce triglyceride levels, modulate inflammatory pathways, and support mitochondrial function^33^. However, the metabolic impact of omega-3 PUFAs may vary by tissue distribution and with age-related physiological changes^34^. Supporting this complexity, a prior study^35^ using the same AusDiab cohort reported that high dietary n-3 intake was associated with increased all-cause mortality. Several factors may underlie this observation. Dietary assessments based on food frequency questionnaires are prone to measurement error. In the Australian context, meat - particularly red meat - also contributes to n-3 intake, which may introduce potential confounding due to the high levels of saturated fat typically present in red meat^36^. A potential contributor to the unusual omega-3 association is the complex interaction with epigenetic ageing, where DNA methylation of *ELOVL2* represents the strongest biological marker of ageing^37–39^. ELOVL2 is required for the elongation of EPA (20:5), ultimately metabolising to DHA after desaturation and a peroxisomal oxidation step, and methylation of ELOVL2 results in reduced expression^40^. Together, these findings highlight the complexity of PUFA biology in ageing and underscore the need for objective lipidomic biomarkers and longitudinal designs to disentangle true biological effects from dietary reporting bias.

Our study identified that an elevated metabolic age was associated with an increased risk of clinical outcomes such as Alzheimer s disease, cardiovascular disease and mortality. Notably, after controlling for environmental factors, EAmAgeΔ consistently showed a stronger association with these clinical outcomes compared to the original unadjusted model. Specifically, for associations with incident Alzheimer’s disease, mAge_origΔ did not reach statistical significance, whereas EAmAgeΔ demonstrated a substantial improvement in both effect size and p-values (Figure 3). These findings demonstrate the improved performance of models that control for environmental factors.

Age and APOE4 are two major risk factors for Alzheimer s disease^41^. After controlling for these factors, our study found that higher metabolic aging (EAmAgeΔ) was significantly linked to an increased prevalent or future risk of AD. To date, published studies on metabolic age have reported weak to no associations between metabolic age and future AD dementia risk^42^, whereas studies on biological ageing measured by DNA methylation^43,44^ have reported significant but very mild associations between their biological ages and future dementia risk. In contrast, independent of age, EAmAgeΔ showed promising prediction ability for the future dementia disease risk (11% hazard increase per EAmAgeΔ year and p value=2.30×10^−05^ in ADNI). To contextualise this effect size, individuals in the highest decile of EAmAgeΔ (≥90th percentile) exhibited approximately two additional metabolic ageing years relative to the population mean in ADNI, underscoring that the observed risk gradients reflect biologically meaningful variation rather than arbitrary stratification. Furthermore, we found that EAmAgeΔ was moderately associated with AD related A/T/N biomarkers, independent of age. Specifically, Higher EAmAgeΔ has been shown to correlate with increased amyloid PET, decreased temporal lobar FDG uptake, and reduced hippocampal volume on MRI. These AD related biomarkers reflect underlying biological process rather than a clinical diagnosis of AD^45,46^. Previous research^47,48^ has demonstrated that the A/T/N classification system, based on these biomarkers, has clinical potential for confirming diagnosis and guiding therapeutic decision-making at an early stage. The significant associations between metabolic age and the A/T/N biomarkers suggest that individuals of the same chronological age but with higher metabolic age may be at higher risk of developing AD, potentially several years before AD symptoms arise.

We also observed that an accelerated metabolic age score was significantly associated with increased risk of cardiovascular disease and all-cause mortality, though such associations were not as strong as with AD risk. Independent of age, a per year increase of EAmAgeΔ was associated with 4%−6% higher hazard for future CVD events. A similar effect size was identified for the associations between all-cause mortality risk and EAmAgeΔ. Individuals in the highest decile of EAmAgeΔ corresponded to approximately more than seven additional metabolic ageing years in AusDiab relative to the population mean, again supporting the biological relevance of the observed associations.

The strengths of this study are the recognition of environmental factors as potential confounders of metabolic age scores and the development of an effective way to remove this confounding in the model development stage. The robustness of our findings is supported by validation across three independent cohorts with diverse age structures and clinical profiles.

Several limitations warrant consideration. Firstly, the lack of an external cohort enriched with various diet and lifestyle data to further evaluate and investigate the interactions between mAge and environmental factors. Secondly, the model was developed using a cross-sectional cohort, which does not directly predict future rates of ageing. This limitation might reduce the model s ability to predict future disease risk, thereby limiting its potential clinical utility. Several advanced studies^2,49,50^ have developed pace of ageing scores using longitudinal biomarkers, offering a promising frontier in this field. Finally, the observed positive association between self-reported fish intake or omega3 fatty acids and metabolic age is counterintuitive and likely reflects residual confounding or dietary misclassification rather than a true adverse biological effect. Future work will integrate lipidomic biomarkers of fish-derived fatty acids and longitudinal analyses to better separate environmental dietary influences from intrinsic metabolic ageing processes captured by EAmAge.

In summary, this study presented a novel lipidomic-based metabolic age score that effectively accounted for environmental confounders. The score demonstrated robust associations with multiple clinical outcomes, supporting its relevance as a clinically informative marker of metabolic health. By quantifying systemic lipidomic dysregulation beyond measured lifestyle exposures, EAmAge provides an integrative and modifiable score of metabolic ageing with potential utility for early risk stratification and personalised intervention across chronic age-related diseases.

## Methods

### Study cohorts.

The Australian Diabetes, Obesity and Lifestyle Study (AusDiab): The AusDiab study served as the primary training dataset for all models. The AusDiab cohort represents a national sample of the Australian population and focuses on studying the prevalence and risk factors associated with obesity, diabetes, cardiovascular disease, and kidney disease. The baseline survey took place in 1999/2000, involving 11,247 participants aged ≥ 25 years. These individuals were randomly selected from six states and the Northern Territory, encompassing 42 urban and rural areas across Australia, using a stratified cluster sampling method^51^. Measurement techniques for clinical lipids including fasting serum total cholesterol, HDL-C, and triglycerides as well as for height, weight, BMI, and other behavioural risk factors have been described previously^51,52^. We utilized all available baseline fasting plasma samples from the AusDiab cohort (n = 10,339) after excluding samples from pregnant women (n =21), those with missing data (n= 277), samples with technical issues (instrument missed injections, n = 19) or whose fasting plasma samples were unavailable (n=591). In addition, 291 participants were excluded due to implausible total energy intake values (females <500 or >3500 kcal/day; males <800 or >4000 kcal/day). The average age of the cohort at baseline was 51.3 years, with a standard deviation (SD) of 14.3 years, and women accounted for 55% of the participants (Table 1).

The Busselton Health Study (BHS): The BHS was utilized as a validation cohort. The BHS is a community-based population study recruited in Western Australia since 1966^53,54^. This cohort contains extensive phenotype data, particularly related to cardiovascular disease (CVD) traits. In our analysis, we included a total of 4,492 subjects from the 1994/95 survey of this ongoing epidemiological study (see Table 1). The mean age of the 1994/95 BHS cohort was 50.8 years, with a standard deviation (SD) of 17.4 years, and women constituted 56% of the participants.

The Alzheimer’s Disease Neuroimaging Initiative (ADNI): The ADNI (adni.loni.usc.edu)^29^ was launched in 2003 as a public-private partnership, led by Principal Investigator Michael W. Weiner, MD. The primary goal of ADNI has been to determine whether serial magnetic resonance imaging (MRI), positron emission tomography (PET), biological markers, and clinical and neuropsychological assessments can be combined to measure the progression of mild cognitive impairment (MCI) and early Alzheimer s disease (AD). For up-to-date information, see www.adni-info.org. The ADNI −1, −2 and -GO is a longitudinal study, recruiting 1,517 individuals over 55 years and following them at intervals of 6–12 months for up to a maximum of 10 years. At baseline, there were originally 1,418 individuals, of which we excluded 25 individuals with missing values (10 missing cognitive scores, 3 missing fasting information, and 12 missing BMI), leaving 1,393 participants. The mean age of the ADNI baseline cohort was 73.6, with a SD of 7.1 years. Women constituted 45% of the cohort.

The Aspirin in Reducing Events in the Elderly study (ASPREE): ASPREE is a large-scale randomized, double-blind, placebo-controlled trial that aimed to evaluate the effects of daily low-dose aspirin on prolonging disability-free survival in 19,114 healthy older adults (aged ≥65 years old)^55–58^. A case-cohort subset was created from the initial pool of 19,114 participants who met the following criteria: individuals with blood samples available from baseline collection, individuals with genotype data, and individuals residing in Australia as baseline. From this subset, 3,287 controls were randomly selected to form the cohort. This sample was enriched with all incident dementia cases (n=465), incident cardiovascular disease cases (n=371), and any remaining *APOEε2* and *APOEε4* homozygotes. After excluding 24 participants with failed lipidomic profiles, 17 with missing BMI, and 9 with missing *APOE* genotypes, there remained 3,950 participants. The mean age of the ASPREE cohort was 75, with a standard deviation of 4.4 years. Women constituted 52% of the cohort.

In Table 1, we present the baseline characteristics of participants from all cohorts for comparison. Across the four studies, we observe that the age distribution of AusDiab and BHS were consistent, with a mean age around 50, which is much younger than ADNI and ASPREE where the mean of age of participants was over 70.

### Lifestyle and dietary factors.

In the AusDiab cohort, lifestyle information was collected through interviewer administered questionnaires, providing information on smoking, leisure-time and physical activity. Details of the collection have been described previously^59^. Dietary information was collected using a validated self-administered Anti-Cancer Council semiquantitative food frequency questionnaire (FFQ)^60^. Food intake was reported in grams per day. Nutrient intake was calculated from the FFQ using the NUTTAB95 food composition database^60^. Dietary and nutrient data was rank-based inverse-normal transformed prior to analysis.

### Clinical outcomes.

In the AusDiab cohort, prevalent cardiovascular disease (CVD; n=577) at study entry was defined based on self-reported history of myocardial infarction and/or stroke. Incident cardiovascular events (CVE; n=444) occurring during 12 years of follow-up were prospectively collected and formally adjudicated using hospital records, death certificates, and clinician review. Incident CVE included adjudicated myocardial infarction and adjudicated cerebrovascular events, comprising ischaemic stroke (cerebral infarction) and intracerebral haemorrhage. All-cause mortality (n=1,706) was ascertained through probabilistic linkage with the National Death Index over 17 years of follow-up.

In the BHS cohort, all nonfatal clinical data was collected through health linkage and fatal events were identified via the National Death index. Cardiovascular diseases were defined using the international classification of disease (ICD) codes and included the codes: ICD9: 410-414; ICD10: I20-I25. At baseline, there were 238 prevalent IHD cases and 4,254 controls. Over the 20 years of follow up, 551 IHD events occurred.

The definition of probable AD in ADNI followed the NINDS-ADRDA criteria^61^. In brief, individuals with Mini-Mental State Exam (MMSE) scores between 20 and 26 (inclusive) and a Clinical Dementia Rating Scale (CDR) of 0.5 or 1.0 were classified as probable AD patients^41^. Participants were defined as MCI if they had MMSE scores between 24 and 30, a memory complaint, objective memory loss measured by education-adjusted scores on Wechsler Memory Scale Logical Memory II, a CDR of 0.5, absence of significant levels of impairment in other cognitive domains, and essentially preserved activities of daily living^62^. At baseline, there were 243 prevalent AD and 329 incident AD cases (10 years follow up).

Four AD related biomarkers were available in ADNI^63^: AmyPet (n=742), a global cortical amyloid deposition measured from amyloid PET scans as biomarkers of β-amyloid; pTau (n=1,009), CSF phosphorylated tau (p-tau) levels as the biomarker of fibrillary tau; FDG_Temp (temporal lobar FDG uptake; n=1,059) and HippVol (hippocampal volume MRI; n=1,385), which are both biomarkers of neurodegeneration. All the biomarkers are available in the LONI online portal (https://ida.loni.usc.edu/). Descriptive statistics of these biomarkers is detailed in Supplementary Table 9.

Incident dementia in the ASPREE cohort was defined for participants as a Modified Mini-Mental State (3MS) test score <78 (explaining ≈50% of the dementia diagnosis); a drop of >10.15 points from the predicted score based on their own baseline 3MS adjusted for age and education; a report of memory concerns or other cognitive problems to a specialist; clinician diagnosis of dementia as indicated in the participant’s medical records; and prescription of a cholinesterase inhibitor^62,64^. Incident dementia was defined using a composite set of criteria, rather than relying on a single measure. There were 270 incident dementia cases with 6.5 years follow up in the ASPREE cohort. Additionally, all-cause mortality over 10 years of follow-up (n=468) was included.

More details of the clinical outcomes in different cohorts can be found in the Supplementary Figure 1.

### Lipid extraction and lipidomic profiling.

Lipid extractions and lipidomic profiling were performed using the same platform across all cohorts, with minor differences, and detailed in prior publications^29,65,66^. Lipid extractions were performed using a single-phase extraction, as previously described^67^. In brief, 10 μL of plasma/serum was mixed with 100 μL of butanol:methanol (1:1) containing 10 mM ammonium format and a standard mix of internal standards (Supplementary Table 10–12). Samples were vortexed thoroughly and sonicated for 60 minutes in a water bath maintained at room temperature. Samples were centrifuged at 14,000 g for 10 minutes and the supernatant transferred into sample vials with glass inserts. Samples were stored at −80 °C prior to analysis.

Targeted lipidomic profiling was performed using liquid chromatography electrospray ionization tandem mass spectrometry (LC-ESI-MS/MS). An Agilent 6490 triple quadrupole (QQQ) mass spectrometer was used for AusDiab, BHS and ADNI, while an Agilent 6495C QQQ mass spectrometer was used for ASPREE. Analytical conditions for the mass spectrometers are detailed in Supplementary Table 12. Liquid chromatography was performed with an Agilent 1290 Infinity II HPLC system and ZORBAX eclipse plus C18 column (2.1×100mm 1.8μm, Agilent). The solvent system consisted of solvent A) 50% H_2_O / 30% acetonitrile / 20% isopropanol (v/v/v) containing 10 mM ammonium formate and solvent B) 1% H_2_O / 9% acetonitrile / 90% isopropanol (v/v/v) containing 10 mM ammonium formate. Profiling in ADNI and ASPREE additionally included 5 mM medronic acid in solvent A. A stepped linear gradient was used with a 12.9–16 minute analytical time. Analytical gradients are outlined in Supplementary Table 14. A 1 L sample injection was used for all cohorts. Full technical details of the methodology and transitions/chromatograms are available on our laboratory website: https://metabolomics.baker.edu.au/method/.

Lipid species were monitored using dynamic/scheduled multiple reaction monitoring (MRM). Detailed MRM transitions, parent ions, collision energies and internal standards are shown in Supplementary Tables 10–12. In the AusDiab cohort, 747 lipid species were measured, covering 38 lipid classes.

For the BHS cohort, 596 lipid species covering 33 lipid classes were quantified; 575 of which were common to the AusDiab cohort (highlighted in Supplementary Table 10). Lipidomic profiling in the ADNI and ASPREE studies were performed using an expanded targeted lipidomic profiling strategy^68^, with the addition of approximately 200 novel lipid species from 17 lipid classes^18^. Overall, there were 781 lipid species from 49 lipid classes reported for the ADNI studies.

The ASPREE study measured 801 lipid species from 48 lipid classes.

### Harmonization of lipidomic datasets.

Harmonization of the lipidomic datasets was performed as previously described ^69^. In brief, National Institute of Standards and Technology-Standard Reference Material-1950 (NIST-SRM-1950) samples run interspersed with cohort data (AusDiab, ADNI, and ASPREE) and used to align lipid concentrations using lipid-specific correlation factors. BHS which did not measure NIST-SRM-1950 at the time of profiling was harmonized to AusDiab using healthy matched sub-cohort based on age, sex, BMI, total cholesterol, HDL-cholesterol, and triglycerides.

We designed our study treating AusDiab as the training cohort and all three others as validation cohorts. To ensure optimal model performance, we aimed to retain the maximum number of shared predictors across cohorts. Accordingly, we identified 575 common lipids between AusDiab and BHS, 667 overlapping lipids between AusDiab and ADNI, and 684 lipids shared by AusDiab and ASPREE (Supplementary Table 10–12).

### Construction of metabolic age scores.

In this study, we used AusDiab as the training cohort to build metabolic age models while treating BHS, ADNI and ASPREE as three external validation cohorts. In AusDiab, we randomly split the cohort into 10 folds to create a 10-fold cross-validation framework, where we iteratively selected one fold as the validation set and combined all other folds as the training set.

To construct a metabolic age score that is free of environmental confounding, we leveraged our original metabolic age score (mAge_orig) previously published by our group^13^. This score employed ridge regression^70^ to model the associations between age and the lipid species, while adjusting for sex and BMI:

(1) $$\mathrm{age}\;=\beta_{0}+\beta_{1}\times sex+\beta_{2}\times BMI+\sum_{k=1}^{p}\;\beta_{k+2}\times l_{k}+\varepsilon$$

Here, p is the number of lipids, $l_{k}$ refers the concentrations of individual lipid species. All the continuous predictors were scaled to zero-mean and unit-variance.

To correct for environmental confounding, we employ a similar approach, except with the distinction of removing environmental from the lipidome before fitting them into model (1), as shown in Figure 1. For this new score with the environmental factor adjustment, we termed it as EAmAge.

Step 1 – A penalized LASSO model was utilised to select the environmental factors which collectively explained the maximum variance of age.

Step 2 - For each individual lipid, we regress out these selected environmental factors.

(2) $$\;l=\beta_{0}+\sum_{j=1}^{m}\;\beta_{j}\times {\;\mathrm{lifestyle}}_{j}+\varepsilon \;\tilde{l}=\beta_{0}+\varepsilon =l-\sum_{j=1}^{m}\;\beta_{j}\times {\;\mathrm{lifestyle}}_{j}$$

Here, m is the number of environmental factors selected; l refers the original concentrations of individual lipid species; $\tilde{l}$ is the residualized lipid species concentrations after adjusted out the selected lifestyle factors.

Step 3 - Construct the model using ridge regularization (using 10-fold cross validation)

(3) $$\mathrm{age}\;=\beta_{0}+\beta_{1}\times sex+\beta_{2}\times BMI+\sum_{k=1}^{p}\;\beta_{k+2}\times \tilde{l_{k}}+\varepsilon$$

Step 4 - Extract the weights β from (3) and apply them to the non-residualized lipid species concentrations to AusDiab and the external cohorts.

(4) $$pAge={\overset{ˆ}{\beta }}_{0}+{\overset{ˆ}{\beta }}_{1}\times sex+{\overset{ˆ}{\beta }}_{2}\times BMI+\sum_{k=1}^{p}\;{\overset{ˆ}{\beta }}_{k+2}\times l_{k}$$

Step 5 – Derivation of metabolic age gap/acceleration (metabolic age delta; EAmAgeΔ). EAmAgeΔ was defined as the residual obtained by regressing predicted age (pAge) on chronological age, capturing the extent to which an individual s metabolic age deviates from that expected for their chronological age. The metabolic age score (EAmAge) was subsequently calculated based on age and EAmAgeΔ:

(5) pAge=β0+β1×age+e′EAmAge=age+e′

Here, e′ represents EAmAgeΔ.

To derive EAmAge in external cohorts, we first calculate pAge using equation 4, then calculate mAge using the AusDiab derived ${\hat{\beta }}_{0}$ and ${\hat{\beta }}_{1}$ from equation 5.

(6) EAmAge^Δ=pAge-βˆ0+βˆ1×ageEAmAge^=age+EAmAgeΔ

As mentioned previously, ADNI and ASPREE exhibit a much older age distribution than AusDiab (Table 1). Therefore, during training, we utilized a subset of AusDiab data (age⩾60) to train and build the mAge model for external validation in ADNI and ASPREE.

### Statistical analysis.

Linear regressions were used to examine the associations of metabolic age scores with lifestyle and dietary factors. Continuous variables underwent rank-based inverse normal transformation prior to analysis and models were adjusted for sex, BMI, and energy intake. For regression against AD-based biomarkers (AmyPet, p-tau, FDG_Temp and HippVol), models were adjusted for sex, BMI, APOE genotypes, omega3 intake, and statins. For HippVol, we additionally adjusted the model for intracranial volume and magnetic field strength. Logistic regression^71^ or Cox proportional hazard regression^72^ (R package survival ) was used to assess the associations between clinical outcomes and metabolic age scores. Models against all-cause mortality and cardiovascular disease were adjusted for sex and BMI. For AD and dementia outcomes, models were additionally adjusted for *APOE* genotypes. The derived odd ratios or hazard ratios were utilised to evaluate the strength of the associations, with p-values indicating the level of significance. Time-to-event associations between environmentally adjusted metabolic age (EAmAge) and clinical outcomes were evaluated using Kaplan Meier survival analyses. Participants were ranked according to EAmAge and stratified into deciles, with survival curves generated for the lowest decile (bottom 10%), middle decile, and highest decile (top 10%). Differences in survival between strata were assessed using Cox proportional hazards models to estimate hazard ratios (HRs) and corresponding p values. Models were adjusted for relevant covariates as described above.

## Supplementary Material

Supplementary Files

This is a list of supplementary files associated with this preprint. Click to download.

• SupplementaryFigures.pdf

## Figures and Tables

**Table 1.** Characteristics of participants from the Australian Diabetes, Obesity and Lifestyle Study (AusDiab), the Busselton Health Study (BHS), Alzheimer’s Disease Neuroimaging Initiative (ADNI), and The Aspirin in Reducing Events in the Elderly (ASPREE).

	AusDiab	BHS	ADNI	ASPREE

#Subjects	10,339	4,492	1,393	3,950

Demographic				
Sex (%male)	4,654 (45.0)	1,976 (44.0)	768 (55.1)	1,901 (48.1)
Age (years, mean±sd)	51.3 (±14.3)	50.8 (±17.4)	73.6 (±7.1)	75.2 (±4.4)
BMI (kg/m^2^, mean±sd)	26.9 (±4.9)	26.2 (±4.2)	26.9 (±4.7)	27.9 (±4.6)

Clinical lipids				
Cholesterol (nmol/L, mean±sd)	5.66 (±1.07)	5.58 (±1.11)	4.98 (±0.95)	5.24 (±0.97)
HDL-C (nmol/L, mean±sd)	1.44 (±0.39)	1.39 (±0.39)	1.53 (±0.37)	1.58 (±0.46)
Triglycerides (nmol/L, mean±sd)	1.28 (±0.92)	1.18 (±0.90)	1.17 (±0.53)	1.32 (±0.66)

Prevalent clinical outcomes at baseline				
Prevalent CVD (%)	577 (5.6)	238 (5.3)	-	0 (0)
Prevalent AD (%)	-	-	243 (17.4)	0 (0)
Future clinical outcomes				
CVD (%)	444 (4.3)^1^	551 (5.3)^2^	-	646 (16.3%)^3^
All-cause mortality (%)	1,706 (16.5)^4^	-	-	468 (11.8)^5^
AD-related Dementia (%)	-	-	329(23.6)^6^	270 (6.8)^7^

1 Future CVEs included ischaemic heart disease and cerebrovascular disease with 12 years follow up in AusDiab.

2 Future IHD is ischaemic heart disease with 20 years follow up in BHS.

3 Future CVDs with 10 years follow up in ASPREE.

4 All cause mortality with 17 years follow up in AusDiab.

5 All cause mortality with 10 years follow up in ASPREE.

6 Alzheimer’s Disease with 10 years follow up in ADNI.

7 Dementia with an average follow-up duration of 6.5 years in ASPREE.

**Table 2.** Prediction performance (*R^2^*) of the mAge models with or without environmental adjustment.

Model		AusDiab	BHS	ADNI	ASPREE
pAge	Original^1^	0.69	0.71	-	-
	Environmental adjustment^2^	0.68	0.74	-	-
	Original (≥60 years)^3^	0.37	-	0.26	0.13
	Environmental adjustment (≥60 years)^4^	0.21	-	0.16	0.11
mAge	Original	0.82	0.85	-	-
	Environmental adjusted	0.83	0.89	-	-
	Original (≥60 years)	0.80	-	0.77	0.82
	Environmental adjusted (≥60 years)	0.90	-	0.92	0.85

1 The original model that was developed without environmental adjustment on the whole population in AusDiab.

2 The model that was developed with environmental adjustment on the whole population in AusDiab.

3 The original model that was developed without environmental adjustment on the subset of AusDiab (age>60).

4 The model that was developed with environmental adjustment on the subset of AusDiab (age>60).
